# Proteome Landscapes of Human Hepatocellular Carcinoma and Intrahepatic Cholangiocarcinoma

**DOI:** 10.1016/j.mcpro.2023.100604

**Published:** 2023-06-22

**Authors:** Xiao Yi, Jiang Zhu, Wei Liu, Li Peng, Cong Lu, Ping Sun, Lingling Huang, Xiu Nie, Shi'ang Huang, Tiannan Guo, Yi Zhu

**Affiliations:** 1 Center for ProtTalks, Westlake Laboratory of Life Sciences and Biomedicine, Key Laboratory of Structural Biology of Zhejiang Province, School of Life Sciences, Westlake University, Hangzhou, Zhejiang, China; 2 Institute of Basic Medical Sciences, Westlake Institute for Advanced Study, Hangzhou, Zhejiang, China; 3 Center for Stem Cell Research and Application, Union Hospital, Tongji Medical College, Huazhong University of Science and Technology, Wuhan, Hubei, China; 4 Key laboratory of Biological Targeted Therapy, The Ministry of Education, Union Hospital, Tongji Medical College, Huazhong University of Science and Technology, Wuhan, Hubei, China; 5 Westlake Omics (Hangzhou) Biotechnology Co, Ltd, Hangzhou, Zhejiang, China; 6 Department of Pathology, Union Hospital, Tongji Medical College, Huazhong University of Science and Technology, Wuhan, Hubei, China; 7 Department of Hepatobiliary Surgery, Union Hospital, Tongji Medical College, Huazhong University of Science and Technology, Wuhan, Hubei, China

**Keywords:** hepatocellular carcinoma, intrahepatic cholangiocarcinoma, proteomics, DIA-MS, machine learning, lipid metabolism, extracellular matrix pathway, biomarker, APOE, PKLR, GALK1

## Abstract

Liver cancer is among the top leading causes of cancer mortality worldwide. Particularly, hepatocellular carcinoma (HCC) and intrahepatic cholangiocarcinoma (CCA) have been extensively investigated from the aspect of tumor biology. However, a comprehensive and systematic understanding of the molecular characteristics of HCC and CCA remains absent. Here, we characterized the proteome landscapes of HCC and CCA using the data-independent acquisition (DIA) mass spectrometry (MS) method. By comparing the quantitative proteomes of HCC and CCA, we found several differences between the two cancer types. In particular, we found an abnormal lipid metabolism in HCC and activated extracellular matrix-related pathways in CCA. We next developed a three-protein classifier to distinguish CCA from HCC, achieving an area under the curve (AUC) of 0.92, and an accuracy of 90% in an independent validation cohort of 51 patients. The distinct molecular characteristics of HCC and CCA presented in this study provide new insights into the tumor biology of these two major important primary liver cancers. Our findings may help develop more efficient diagnostic approaches and new targeted drug treatments.

## Highlights

• Comprehensive proteome landscapes of HCC and CCA.
• The dysregulation of lipid metabolism in HCC.
• The activation of extracellular matrix (ECM)-related pathways in CCA.
• A three-protein classifier distinguishing HCC from CCA.

## Graphical Abstract

**Figure:**
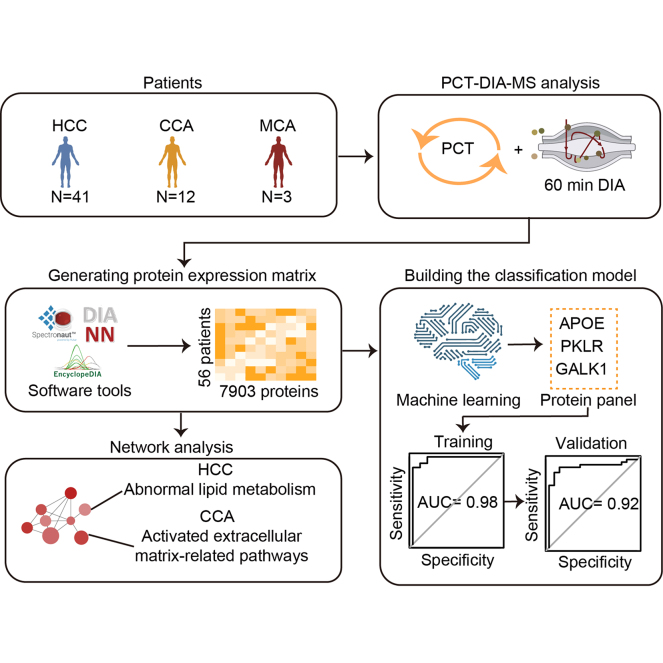

## In Brief

We used the DIA-MS methodology to characterize the proteomes of hepatocellular carcinoma (HCC) and intrahepatic cholangiocarcinoma (CCA). We found an abnormal lipid metabolism in HCC and the activation of ECM-related pathways in CCA. We then developed a three-protein classifier to distinguish CCA and HCC, which achieved an AUC of 0.92 and an accuracy of 90% in the validation cohort. The distinct molecular characteristics of HCC and CCA provided new insights into the tumor biology of liver cancers.

Liver cancer ranked second for its mortality rate according to the cancer statistics of China in 2020 (1). There are three major types of primary liver cancer: hepatocellular carcinoma (HCC), intrahepatic cholangiocarcinoma (CCA), and mixed cell carcinoma (MCA, also known as combined hepatocellular cholangiocarcinoma). Primary HCC is the most common primary liver malignant tumor, accounting for more than 80% of malignant liver tumors (2). CCA is the second most common primary liver malignancy, accounting for 10% to 15% of malignant liver tumors (3). On the other hand, MCA accounts for a minor population of primary liver malignancies.

Surgery is currently the only curative method for HCC. However, most patients with HCC report no symptoms at the early stage of the disease. Therefore, due to the late diagnosis, they lose the opportunity of an optimal surgical resection and have to face a poor prognosis with a 5-year survival rate of only 19.9% (4). Studies have shown that CCA may have a worse prognosis than HCC (2, 4, 5, 6, 7). Surgical resection is the only effective treatment for patients with CCA as well. However, the prognosis of CCA is even worse: the 5-year survival rate is less than 10% (4).

There are significant differences between HCC and CCA. First, hepatitis B and hepatitis C viral infections are important risk factors for HCC, while intrahepatic bile duct calculi are an independent risk factor for CCA. Second, significant heterogeneity has been observed in CCA compared to HCC. This is due to the fact that HCC develops in the context of chronic liver disease, while most CCA develops in normal background livers without any apparent risk factors (8). Additionally, CCA is more aggressive than HCC, and they differ in treatment and prognosis. Specifically, when radical hepatectomy is performed, the resection scope for CCA is more extensive than for HCC (9).

A recent clinical study described the differences in demographics, socioeconomic factors, clinical characteristics, and outcomes in patients with CCA and HCC (4). According to the study, the survival of patients with CCA is shorter than that of patients with HCC, which may be explained by the lack of an adequate surveillance program and the high metastatic potential of CCA (4). Another study identified two common molecular subtypes of patients with CCA and HCC by integrating genomics, transcriptomics, and metabolomics data; one is driven by *PLK1* and *ECT2*, the other by obesity, T-cell infiltration, and bile acid metabolism (10). Despite these discoveries, the pathogenesis and molecular level changes of HCC and CCA are still not fully understood.

Proteomics has shown promising potential for exploring the molecular features of liver cancer. Jiang *et al.* (11) presented three proteomic subtypes of early-stage HCC and identified SOAT1 as a novel therapeutic target for S-III, the least prognostic HCC subtype. Gao *et al.* (12) used proteogenomics to classify hepatitis B virus (HBV)-associated HCC into three subgroups with distinct molecular characteristics relative to metabolic reprogramming, microenvironmental dysregulation, and cell proliferation, leading to different clinical and therapeutic approaches. They also found that *CTNNB1* mutation-associated ALDOA phosphorylation promotes HCC cell proliferation (12). Another study used proteomic-based profiling to identify four subgroups of patients with intrahepatic CCA, which have unique characteristics in terms of the genome, the immune microenvironment, the therapeutic strategy, and the clinical prognosis (13). They also characterized five major driver genes for intrahepatic CCA (*i.e.*, *TP53*, *KRAS*, *FGFR2*, *IDH1/2*, and *BAP1*), which could be explored as novel therapeutic targets (13). Mutations in these five driver genes play roles in biological processes such as cell cycle, drug metabolism, and inflammation infection (13). Together, these studies provide several clues for understanding the tumor biology of HCC and CCA. However, a comprehensive comparison of the proteomics landscapes of these two cancer types remains elusive.

In this study, we first performed DIA-based proteomics analysis of HCC and CCA tissue samples. We then used this data to investigate the proteomes of HCC and CCA. We found two distinct molecular signatures: the activated ECM-related pathway for CCA and the activated lipid metabolism pathway for HCC. The unique molecular features and pathways uncovered could contribute to the different pathogenesis and prognosis features of HCC and CCA. Finally, based on our data, a machine learning model was trained and used to successfully distinguish HCC from CCA.

## Experimental Procedures

### Declaration of Helsinki Principles

This work was conducted according to the Declaration of Helsinki's ethical principles. The liver samples were obtained from Union Hospital of Wuhan in China and Outdo BioTech of Shanghai in China with the approval of the ethics committee of Tongji Medical College, Huazhong University of Science and Technology (Permission number: S018) and Westlake University (Permission number: 20190312GTN0005).

### Experimental Design and Statistical Rationale

In this study, we recruited 107 liver cancer patients in two cohorts. A total of 303 MS data for 163 liver tissue samples were acquired, including 54 data-dependent acquisition (DDA) runs, 54 information-dependent acquisition (IDA) runs, 138 DIA runs, and 57 sequential window acquisition of all theoretical spectra (SWATH) runs. The DDA and IDA data were utilized for spectral library construction for parsing DIA and SWATH data, respectively.

As to spectral library establishment, a pooled liver peptide mixture from all the samples was pre-fractionated either by strong cation exchange into 14 fractions or by high pH HPLC separation into 40 fractions before they are subjected to DDA or IDA analysis (54 MS runs each), as previously described with small changes (14).

As to quantitative DIA proteomics analysis,112 peptide samples were obtained from 56 paired fresh frozen (FF) tumor tissues and adjacent benign tissues, from the first cohort of 56 patients including 41 HCC, 12 CCA, and 3 MCA cases. Besides, 26 peptide samples were randomly selected as technical replicates for DIA-MS acquisition. Therefore, 138 DIA-MS runs were performed.

As to quantitative SWATH proteomics analysis, 51 peptide samples were obtained from formalin-fixed, paraffin-embedded (FFPE) tissue blocks from the second patient cohort including 34 HCC and 17 CCA cases. In addition, six peptide samples were randomly selected as technical replicates for SWATH-MS acquisition. Therefore, 57 SWATH runs were acquired.

### Patients and Tissue Samples

The liver samples were collected from the training and validation cohorts. For the training cohort, 56 patients with liver cancer were enrolled from Union Hospital, Tongji Medical College, Huazhong University of Science and Technology, Wuhan, China. All tissue samples were assessed by the histomorphological examination and collected from hepatectomy specimens within 1 h after the surgical removal, snap frozen, and stored at −80 °C till the proteomics analysis. For the validation cohort, 51 FFPE tumor tissues from 51 patients with liver cancer were obtained from Outdo BioTech (Shanghai, China). Detailed information of all the patients is provided in supplemental Tables S1 and S2.

### PCT-Assisted Sample Preparation and DIA/SWATH-MS Data Acquisition

The tissue samples were processed for proteomic analysis as previously described (15, 16, 17). For the training cohort, 112 FF tissues were processed into peptides using the pressure cycling technology (PCT)-assisted method. Briefly, the FF tissue sample was lysed with lysis buffer (6M urea, 2M thiourea in 0.1 M ammonium bicarbonate) using PCT. The extracted protein solution was reduced and alkylated by incubating with 10 mM tris(2-carboxyethyl)phosphine and 20 mM iodoacetamide. Then proteins were digested by Lys-C (enzyme-to-substrate ratio, 1:40) and Trypsin (enzyme-to-substrate ratio, 1:50) using PCT-assisted digestion. Peptide samples were then acidified by trifluoroacetic acid and cleaned by C18 desalting. For the validation cohort, the FFPE tissue was firstly de-paraffinized, re-hydrated and hydrolyzed, and was then followed by the PCT-assisted peptide preparation procedure as described above for FF tissues.

### DIA-MS Data Acquisition

For the training cohort, the DIA mode was utilized for the MS data acquisition. The detailed procedure for deriving DIA data has been previously described (18). In brief, each DIA acquisition involved analyzing 0.5 μg of the peptide using a nanoflow DIONEX UltiMate 3000 RSLCnano System (Thermo Fisher Scientific) coupled with a Q Exactive HF hybrid Quadrupole-Orbitrap (QE-HF, Thermo Fisher Scientific). Peptides were separated by a 60-min liquid chromatography (LC) gradient (from 3% to 28% buffer B; where buffer A was composed of 98% H_2_O, 2% ACN, and 0.1% FA, and buffer B was composed of 98% ACN, 2% H_2_O, and 0.1% FA). MS1 was performed in the m/z range of 390 to 1210 with a resolution of 60,000, automatic gain control (AGC) target of 3e6, and a maximum ion injection time of 80 ms. MS2 was performed with a resolution of 30,000, an AGC target of 1e6, and a maximum ion injection time of 50 ms. DIA-MS used 24 isolation windows.

For the validation cohort, the SWATH mode was employed for the MS data acquisition. The detailed procedure for deriving SWATH data has been previously described (19). In brief, each SWATH acquisition involved analyzing 2 μg of the peptide using an Eksigent 1D+ Nano Liquid Chromatography (LC) system (Eksigent) coupled to a 5600 TripleTOF mass spectrometer (SCIEX). The LC gradient was 60 min (from 5% to 30% buffer B; where buffer A and buffer B were the same as described above for QE-HF analysis). The SWATH acquisition was performed on 66 variable windows, with an ion accumulation time of 40 ms for each SWATH window and 50 ms for peptide precursors.

### Establishing Spectral Libraries for DIA-MS

In total, 54 DDA-MS data were used to build the spectral library for subsequent quantitative DIA analysis. Two libraries were built using the spectrum-centric approach. The first was built using Spectronaut (version 14.3.200701.47784), which was commercial software (20). The parameters were set by default, with carbamidomethyl (C) designated as the fixed modification and oxidation (M) as the variable modification. Two missed trypsin cleavages were permitted, and the precursor and fragment tolerance were set as dynamic. Two calibration searches were conducted to determine the ideal tolerance for the second-pass calibration, with a rough calibration based on the first-pass calibration and the finer calibration based on the second-pass calibration. The thus built-up library was then optimized using the SubLib method (21), resulting in a library consisting of 114,890 peptides, 9074 protein groups, and 8962 proteins. The second one was built using open-source software, OpenSWATH (version 2.5.0) (22) and pFind (version 3.1.5) (23), as an alternative for cost-free DIA analysis. It contained 93,485 peptides, 10,863 protein groups, and 8834 proteins, and was further optimized using the SubLib method for DIA-NN (version 1.7.5) (24), and EncyclopeDIA (version 0.9.0) (25), respectively.

As to SWATH, 54 IDA-MS data were employed, using the spectrum-centric approach to build the spectral library for subsequent quantitative proteomics analysis. OpenSWATH (version 2.5.0) and MaxQuant (version 1.6.14.0) (26) were used to build the spectral library. The library was then optimized using the SubLib method for DIA-NN analysis. The resulting library consisted of 46,110 peptides, 9044 protein groups, and 7657 proteins.

All three libraries were searched against a Swiss-Prot protein database (downloaded on 15 July 2020, containing 20,368 reviewed protein sequences), with a precursor and protein identification false discovery rate (FDR) cutoff of 0.01.

### DIA-MS Data Analysis

As described above, library-based DIA data analysis was conducted using Spectronaut, DIA-NN, and EncyclopeDIA, all of which are state-of-the-art software tools for quantitative proteomic analysis. DIA/SWATH data sets were analyzed using the peptide-centric approach. To ensure high-quality results, we employed rigorous parameters in our data analysis. For Spectronaut, we set the RT prediction type to dynamic iRT and used dynamic MS mass tolerance. Peptide identification required at least three fragment ions, and major and minor group quantities were based on mean peptide and mean precursor quantity, respectively. The protein quantification was based on the mean value of the top three intensities of proteotypic peptides. In DIA-NN, automatic mass and RT correction were performed, and the top six fragments (ranked by their library intensities) were used for peptide identification and quantification. The protein intensities were calculated as the sums of the intensities of the top three most abundant precursors identified. For EncyclopeDIA, the precursor, fragment, and library mass tolerance were set as 10 ppm. The RT model was generated from peptides detected at 1% FDR using a nonparametric kernel density estimation algorithm that follows the density mode over time. Peptides were quantified using the sum of the top five transitions, and protein quantities were calculated as the sum of peptide quantities. All software was set to 1% FDR at the peptide precursor level to ensure high confidence in the results.

The protein and peptide quantification matrices obtained from each software are provided in supplemental Table S3, with all MS intensity values log2 transformed for consistency and ease of interpretation.

### Differential Expression Analysis

Before performing the differential expression analysis, we removed the proteins that were not detectable in more than 90% of the samples; the missing protein abundance values were imputed as zeros. The protein fold change (FC) between the two groups was computed as the ratio of the mean protein abundances in each group. The *p*-values were calculated using the two-sided paired Welch’s *t* test between the tumor tissues and their matched benign tissues. For the analysis of HCC and CCA tumor tissues, the *p*-values were calculated using the two-sided unpaired Welch’s *t* test. All the *p*-values underwent a Benjamini-Hochberg correction (adjusted *p*-values). Proteins with adjusted *p*-values <0.05 and absolute |log_2_(FC)| > 0.5 were considered significantly changed proteins.

### Network Analysis

An IPA (27) was used to identify the pathways that involve the differently expressed proteins between tumor and benign tissues. First, the proteins’ log_2_(FC) values were inputted as the observation values. Then, the pathways’ *p*-values from our IPA analysis were calculated using the right-tailed Fisher's exact test.

**Fig. 1:**
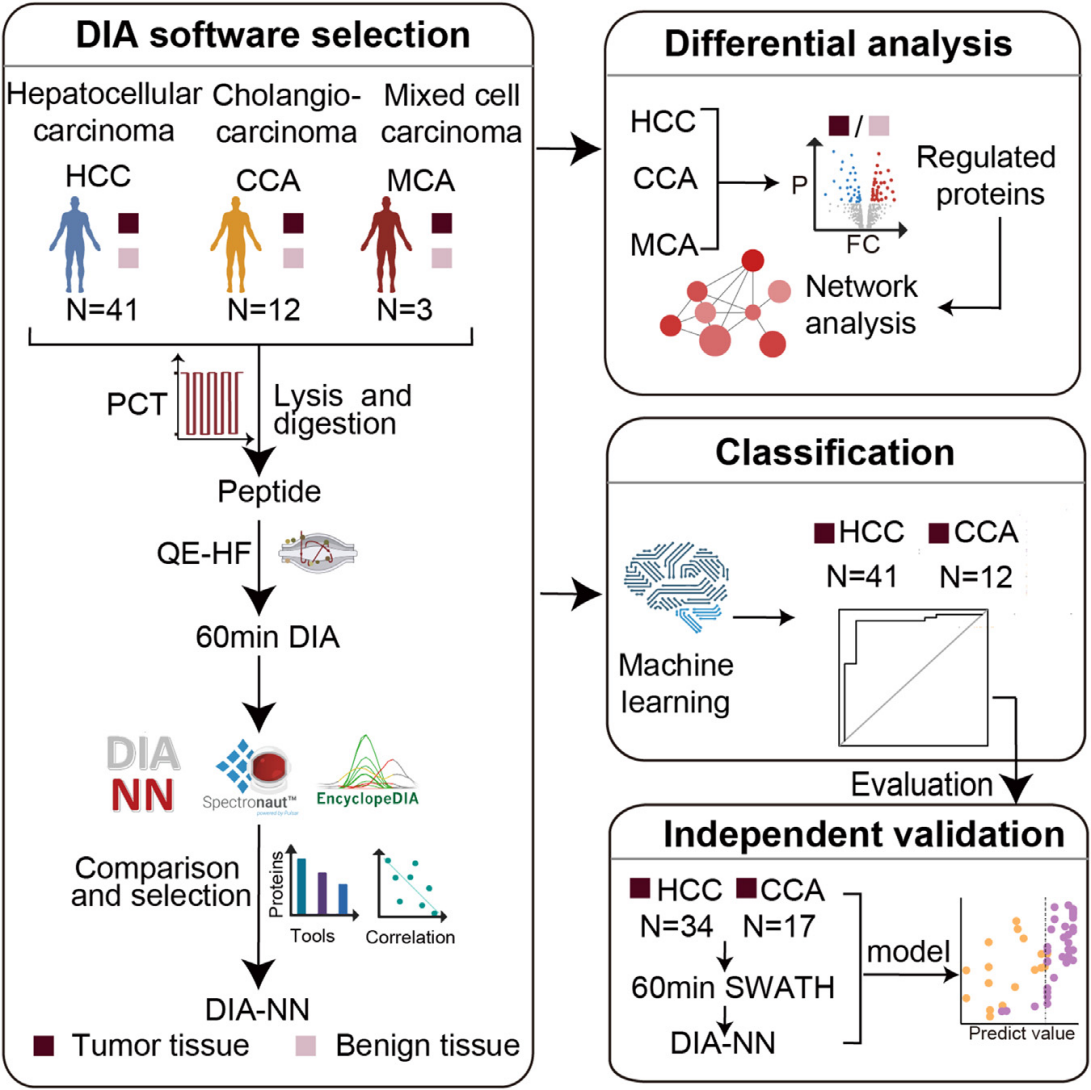
**Study design and data analysis pipeline.**

**Fig. 2:**
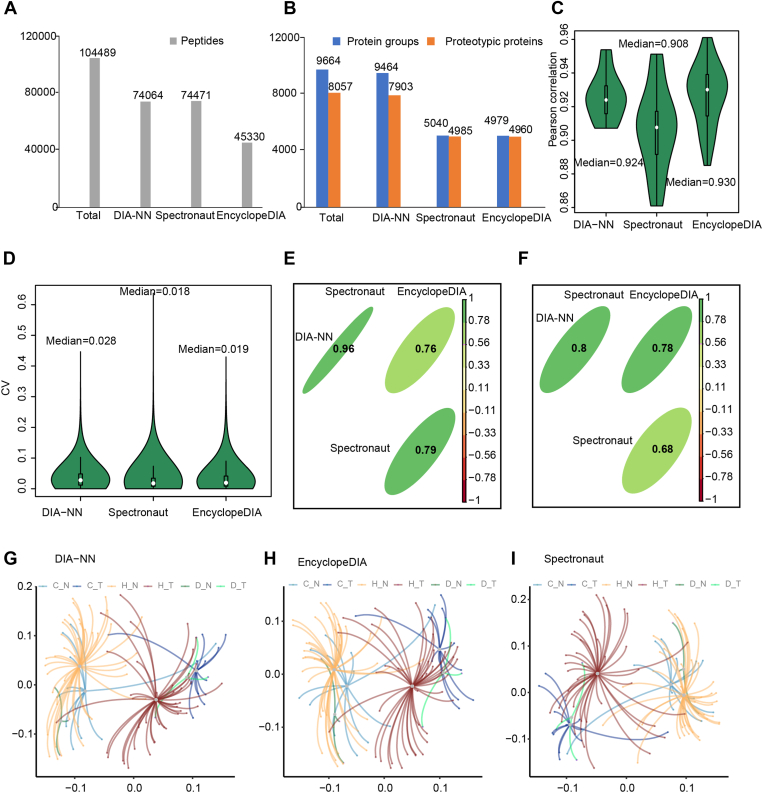
**DIA raw data analysis.***A*, number of peptides identified using three DIA software tools. *B*, number of proteins identified by three DIA software tools. *C*, violin plot showing the Pearson correlation coefficients of the protein intensities of our 26 technical replicate pairs computed with three DIA software quantifications. *D*, coefficients of variation (CV) of the proteins’ quantification for 26 technical replicate pairs. The *black boxes* and *white dots* represent the lower quartile, upper quartile, and median values, respectively. *E* and *F*, the Spearman correlation coefficients of the intensities of 34,214 overlapped peptides (*E*) or 4348 overlapped proteins (*F*) in the three DIA software. *G*–*I*, based on the three protein quantification results derived from DIA-NN, EncyclopeDIA, and Spectronaut, the three corresponding PCA plots show the distribution of benign (N) and tumor (T) samples belonging to the HCC (*H*), CCA (*C*), and MCA (*D*) tumor subtypes using the first two principal components.

**Fig. 3:**
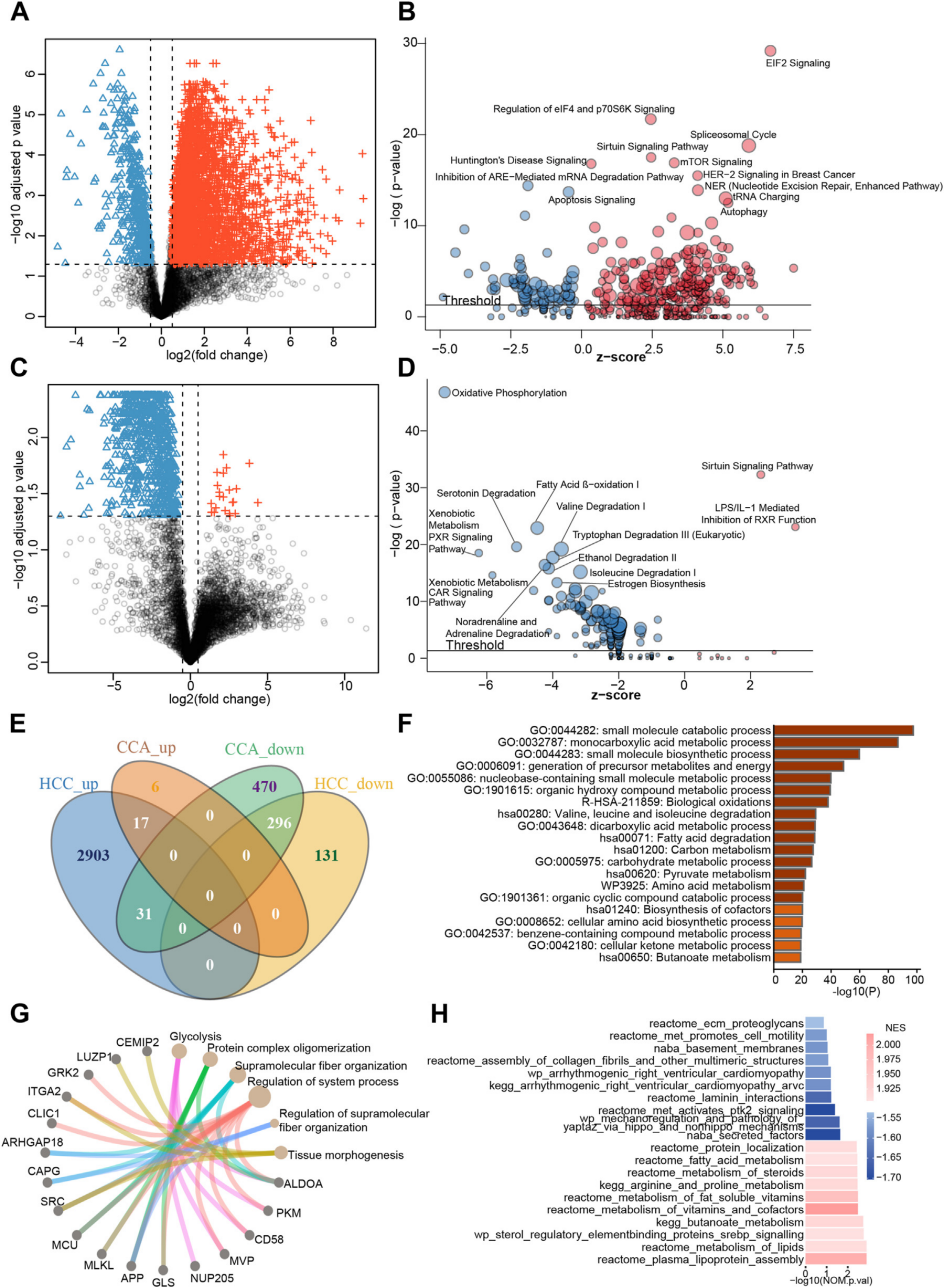
**Comparison of the regulated proteins between the tumor samples and the adjacent benign tissues from 41 patients with HCC and 12 patients with CCA.***A* and *C*, volcano plots showing the proteins with significant abundance differences between tumor and benign tissues in HCC (*A*) and CCA (*C*) samples. Those proteins with a |log_2_(FC)| > 0.5 and an adjusted *p*-value <0.05 (BH method) between the two groups were considered significantly dysregulated. The up-regulated proteins in the tumor tissues are highlighted in *red*, the down-regulated ones in *blue*. *B* and *D*, the most significantly changed pathways were enriched using IPA and the dysregulated proteins for 41 HCC (*B*) and 12 CCA (*D*) patients. *E*, Venn diagram showing the overlapping dysregulated proteins between HCC and CCA. *F* and *G*. pathways involving the overlapping down-regulated (*F*) or up-regulated (*G*) proteins between HCC and CCA (enrichment analysis performed using Metascape). *H*, the ten most enriched pathways assigned by GSEA to HCC and CCA.

The pathways involving the proteins that were significantly regulated in both CCA and HCC tissues (Fig. 3, *E*–*G*) or the differentially expressed proteins in the HCC or the CCA tumor tissues (supplemental Fig. S5) were annotated using Metascape (https://metascape.org/) (28) with the default method.

To investigate the differences between the HCC and the CCA tumor tissues, we performed a pathway enrichment analysis using GSEA software (v4.1.0) (29) (Fig. 3*H* and supplemental Fig. S4, *A* and *B*). The protein abundances from these two groups were inputted as an expression dataset. The gene set database used in our analysis was “c2.cp.v7.2.symbols” and the number of permutations was set to 1000. The normalized enrichment scores and the nominal *p*-value estimates were the primary statistics for evaluating our gene set enrichment results.

### Machine Learning

**Fig. 4:**
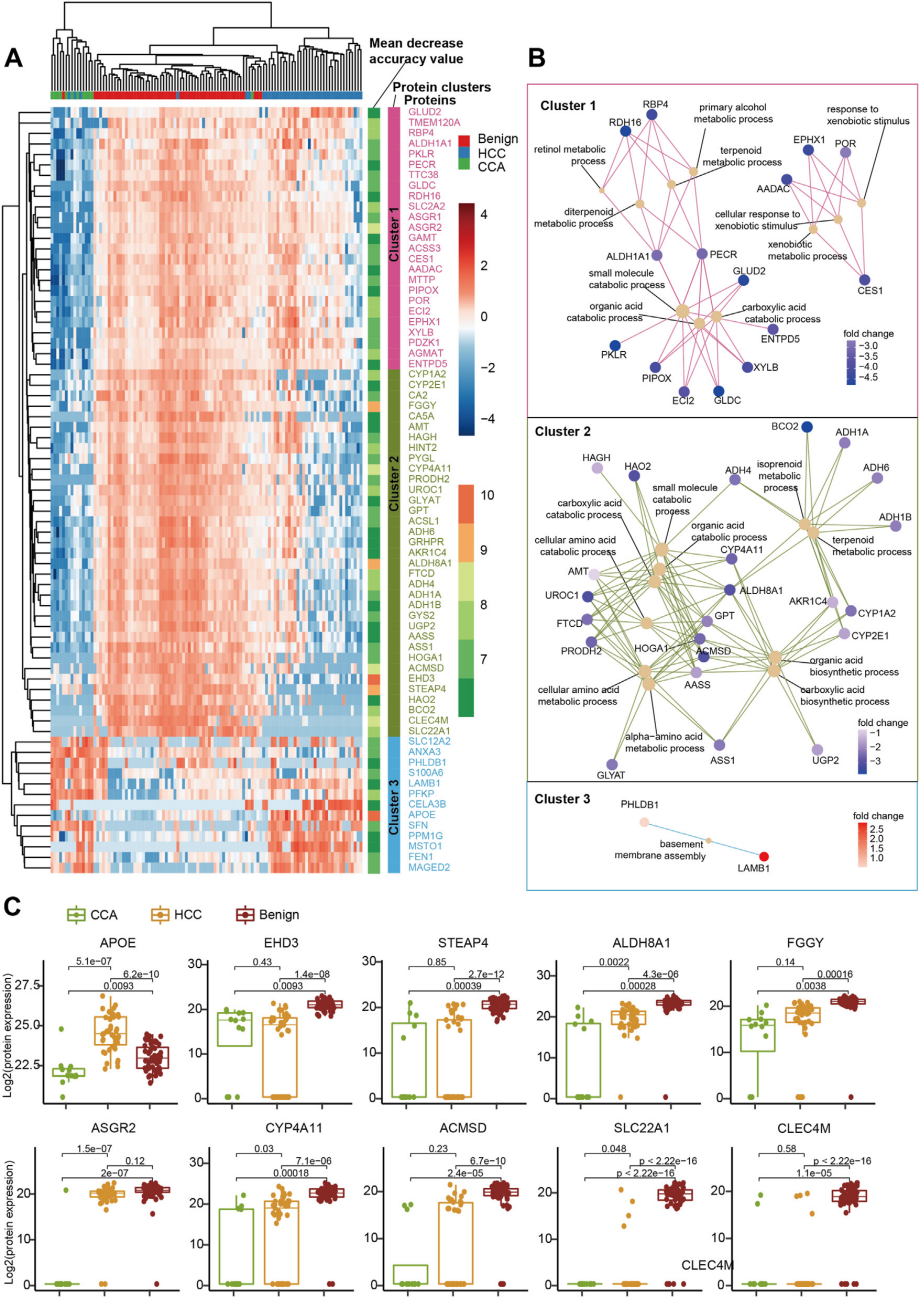
**Machine learning-based classification of the HCC tumor tissues, the CCA, tumor tissues, and the benign tissues.***A*, the top 73 protein features, ranked by the mean decrease in accuracy, were selected through a random forest analysis. These proteins were unsupervised clustered into three clusters based on protein abundance, and were visualized with a heatmap. *B*, the biological process involved in the top 73 protein features annotated using the Gene Ontology database and visualized using the R package clusterProfiler. *C*, boxplot shows the top ten proteins, which ranked highest in the mean decrease in accuracy, in the CCA tumor tissues, the HCC tumor tissues, and the adjacent benign tissues.

An R package, RandomForest (version 4.6.14), was used for the features’ selection and to build a classification model. For the feature selection of HCC, CCA, and benign tissues (Fig. 4*A*), the “ntree” were set to 1000, and the node size was set to 1. The cutoff of the protein mean decrease accuracy was set to 6.

Before building the classification model, we selected the model’s features using the random forest algorithm with the training cohort. We then selected the 15 proteins that ranked highest in the mean decrease in accuracy. The missing values of the features were complemented using the k-nearest neighbors' algorithm (30). By arbitrarily combining these 15 features, we built 2ˆ15 − 1 models using the random forests algorithm, building each model by setting the “ntree” to 1000 and the node size to 1. We finally evaluated the performance of these models using a 5-fold cross-validation and selected the model with the highest AUC value.

## Results and Discussion

### Study Design and Workflow

We enrolled a cohort of 56 patients with liver cancer, including 41 HCC, 12 CCA, and 3 MCA cases. Their detailed clinical and pathological data are shown in supplemental Tables S1 and S2. Two biopsies were obtained from each patient: one derived from the tumor tissue and the other from the adjacent benign tissue, as assessed by the histomorphological examination. First, all the samples underwent a PCT-based peptide extraction and a subsequent 60 min DIA-based MS data acquisition. We used three broadly used software (*i.e.*, Spectronaut (20), DIA-NN (24), and EncyclopeDIA (25)) to parse our DIA data. As DIA-NN demonstrated superior performance in our data, the protein quantification results from DIA-NN were used in our subsequent analysis. We then used our quantitative proteomics data to explore the differences between the tumor and benign samples of our HCC and CCA patients. Finally, a machine-learning classification model was built to distinguish CCA from HCC tumors. The performance of the model was validated in a second independent cohort (Fig. 1).

### DIA-MS Data Analysis and Quality Control

From our first cohort of 56 liver cancer patients, 112 peptide samples were generated and analyzed using DIA-MS. To minimize the batch effects, all samples were randomly shuffled during the sample preparation and the MS acquisition. In addition, for quality control purposes, 26 samples were randomly selected from 112 peptide samples as technical replicates. In total, 138 DIA-MS runs were performed.

It is well known that DIA data are naturally complex because of the loss of direct relationship between peptide precursors and their fragment ions, and multiple computational tools have been developed to analyze them (31). Spectronaut is a commercial software that develops a hybrid-library-dependent approach to further resolve the imprecision in retention time for different chromatography and loss of FDR control at the protein and peptide level. DIA-NN is an open-source software that uses deep neural networks to distinguish real signals from noise and explores new quantification and signal correction strategies for the processing of DIA data. EncyclopeDIA is also an open-source software that improves peptide identification by calibrating retention times and fragment specificity in chromatographic libraries. However, due to algorithm differences, the same dataset parsed with different software tools gave partially different quantitative results (32).

We identified and quantified 104,489 peptides and 8057 proteotypic proteins (Fig. 2, *A* and *B* and supplemental Table S3) using three DIA tools as described above. Of these, 34,214 peptides and 4348 proteotypic proteins were consistently quantified by all three DIA software tools (supplemental Fig. S1, *A* and *B*).

To evaluate the quality of our MS data, we calculated the Pearson correlation coefficients (Pearson r) and the coefficients of variation (CVs) between the protein intensities from each pair of technical replicates generated by the three software tools. As a result, all 26 technical replicate pairs showed strong correlations (Pearson r > 0.9) (Fig. 2*C*) and low CV values (median CV ∼ 2%) (Fig. 2*D*) among all three DIA software tools. These results showed a high degree of reproducibility of our data.

To further check the correlation of the quantitative proteomes generated using different DIA software tools, we calculated Spearman’s rank correlation coefficients (Spearman *r*) for the 34,214 peptides and 4348 proteins identified by all three tools. We found that DIA-NN and Spectronaut showed the strongest correlation (Spearman *r* > 0.8) at both the peptide and protein quantification levels (supplemental Fig. S2, *E* and *F*). Using these commonly quantified proteins, we further performed principal component analysis (PCA) to distinguish tumor samples from the adjacent benign samples for all patients. As shown by PCA results, tumor tissues and the adjacent benign tissues from all patients could be distinctly clustered into two groups (supplemental Fig. S2, *G*–*I*). While the DIA-NN results showed a robust correlation with the other two software tools and identified more proteins (Fig. 2, *B*, *E*, and *F*). Therefore, for our subsequent analyses, we used the protein quantification data matrix generated by DIA-NN (Fig. 1).

### Different Proteome Landscapes and Characteristics of HCC and CCA

To identify which proteins are specifically expressed by each liver tumor type, we performed a differential expression analysis between the data from the tumors and the benign tissues. In particular, we selected those proteins with an abundance difference of |log_2_(FC)| > 0.5 and with an adjusted *p*-value <0.05 (Benjamini-Hochberg method) between tumor and benign tissue (supplemental Table S4). However, as we did not find any significantly regulated proteins between the MCA tissues and their corresponding benign samples (supplemental Fig. S2*A*), we did not further analyze this tumor subtype; in our subsequent analyses, we focused on the proteomes of HCC and CCA.

A total of 3378 proteins were significantly dysregulated in the HCC samples compared with their adjacent benign tissues; in particular, 87% of these proteins (2951 out of 3378) were up-regulated in the tumor samples (Fig. 3*A*). We next performed an ingenuity pathway analysis (IPA) to find which pathways were affected by the differentially regulated proteins. We found that most pathways had positive Z-score values, indicating they were activated. These activated pathways were mainly involved in transcription, mRNA translation regulation, and post-translational modification; they included EIF2 signaling, the regulation of eIF4E and p70S6K signaling, the spliceosomal cycle, sirtuin signaling, mTOR signaling, and tRNA charging (Fig. 3*B*).

We next analyzed the CCA dataset. A total of 820 proteins were significantly dysregulated in the CCA samples compared with their adjacent benign tissues. Unlike in HCC, most of the proteins regulated in the CCA tumor tissues were down-regulated (97% of 820 proteins) (Fig. 3*C*). The IPA enrichment of these proteins showed that most regulated pathways were inhibited in the CCA tissues. These pathways were mainly associated with liver metabolic processes, such as oxidative phosphorylation, fatty acid β-oxidation I, serotonin degradation, valine degradation I, and xenobiotic metabolism PXR signaling (Fig. 3*D*).

Next, we compared the differentially expressed proteins between the HCC and the CCA groups. Our HCC and CCA tumor samples shared 296 down-regulated proteins and 17 up-regulated ones (Fig. 3*E*). We then performed a pathway enrichment analysis of these shared proteins using Metascape (28). We found that the 296 down-regulated proteins were mainly involved in pathways related to small molecule metabolic processes (Fig. 3*F* and supplemental Fig. S2, *B* and *C*); the 17 up-regulated proteins were involved in processes such as system progress, supramolecular fiber organization, and protein complex oligomerization (Fig. 3*G*).

To investigate the differences between HCC and CCA, a gene set enrichment analysis (GSEA) was conducted between the HCC and the CCA tissues (Fig. 3*H*). We found that extracellular matrix (ECM)-related pathways were significantly activated in the CCA tumor tissues, including secreted factors, MET activates PTK2 signaling, laminin interactions, assembly of collagen fibrils and other multimeric structures, basement membranes, and MET promotes cell motility (Fig. 3*H*). This finding may explain the high degree of malignancy, the easy involvement of peripheral organs, and the distant metastasis formation of CCA since the up-regulation of ECM-related protein expression is associated with tumor metastasis formation and invasion (33, 34, 35). Additionally, we found that in the HCC tumor tissues, the most significantly enriched pathways were mainly related to lipid metabolism, such as plasma lipoprotein assembly, metabolism of lipids, sterol regulatory element-binding proteins (SREBP) signaling, metabolism of vitamins and cofactors, metabolism of fat-soluble vitamins, metabolism of steroids, and fatty acid metabolism (Fig. 3*H*). Notably, lipid metabolism reprogramming can provide energy for HCC cell proliferation (36). Furthermore, HBV, hepatitis C virus, aflatoxin, and excessive alcohol and tobacco consumption are known as the main causes of HCC. They act independently or synergistically to cause lipid degeneration and lipid deposition in liver cells and promote malignancy (36). Also, our results complement the findings of HCC-related transcriptomics studies that found differences in the transcript levels of genes associated with lipid metabolism (37).

### Key Proteins Distinguishing HCC, CCA, and Benign Tissues

To classify tissue samples as belonging to HCC, CCA, or benign tissue, we identified 73 key proteins using a random forest-based feature selection. An unsupervised cluster analysis of HCC, CCA, and benign tissue based on the 73 proteins showed that most samples clustered according to their pathological type, suggesting that these proteins could be used to distinguish the three tissue types (Fig. 4*A*). Also, the proteins of each cluster were enriched in specific biological processes: small molecule metabolic process, lipid metabolic process, and basement membrane assembly (Fig. 4, *A* and *B*). The top ten featured proteins which ranked by mean decrease accuracy were shown in Figure 4*C*, and their functions are described in the Supplemental Information.

### Machine Learning-Based Classification of HCC and CCA Tumor Tissues

**Fig. 5:**
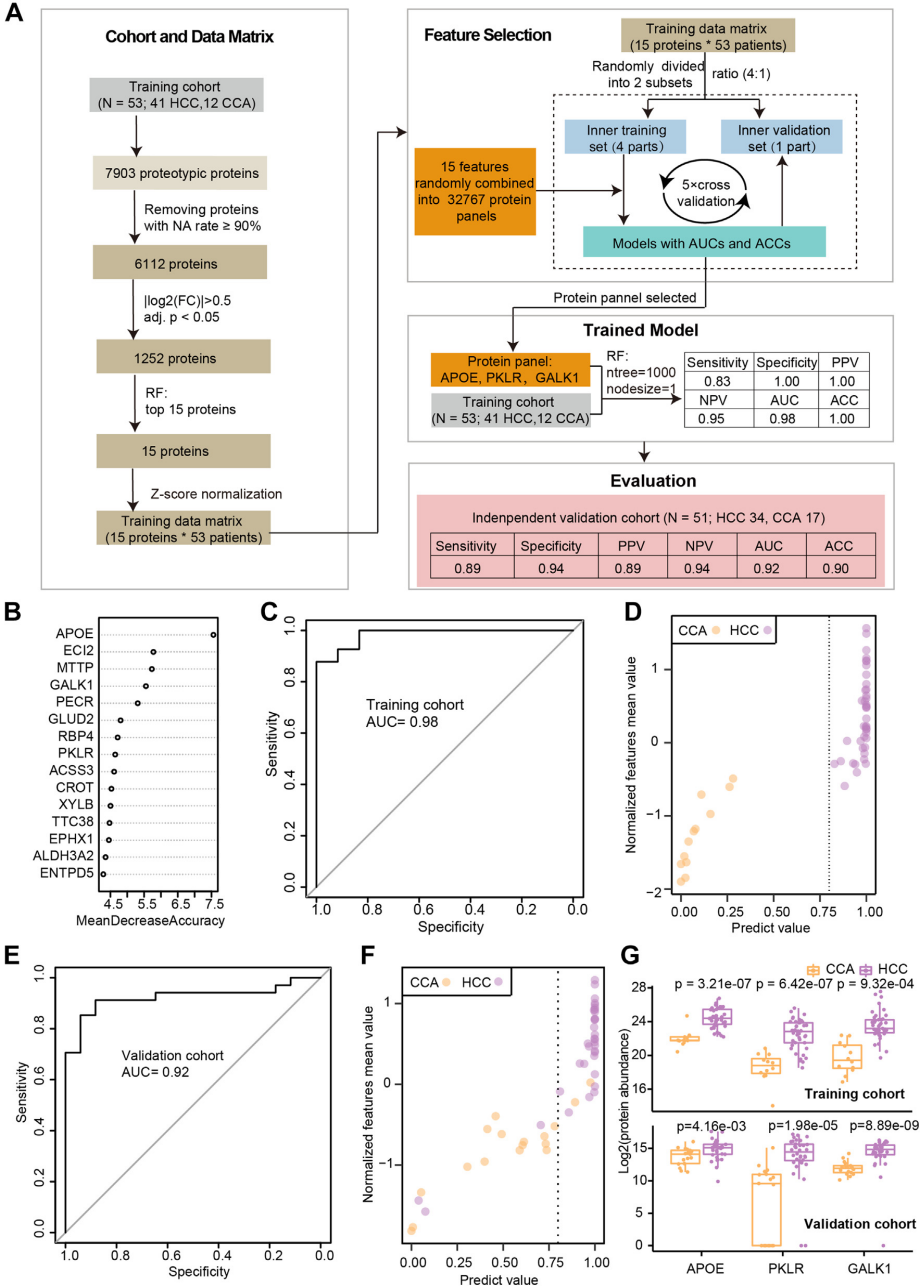
**Machine learning-based classification of HCC and CCA tumor tissues.***A*, workflow for generating a model to distinguish HCC and CCA tumors. *B*, fifteen proteins were prioritized by our machine learning model. *C*, receiver–operating characteristic (ROC) curves of the random forest model in the training cohort. *D*, performance of the three selected proteins with the training cohort of 41 HCC and 12 CCA samples. *E*, ROC curves of the random forest model in the validation cohort. *F*, performance of the three selected proteins with the validation cohort of 34 HCC and 17 CCA samples. *G*, boxplots showing the expression of the three selected proteins in HCC and CCA in the training cohort and validation cohort.

We next developed a machine learning classification model to distinguish HCC and CCA tumors (Fig. 5*A*). We established strict inclusion criteria and excluded participants who were not diagnosed with HCC or CCA. Our training cohort included 41 HCC and 12 CCA cases. To ensure data quality, we removed proteins with a high proportion of missing values (NA rate ≥ 90%). We then took into account those proteins with *p*-values <0.05 and |log_2_(FC)| > 0.5 between the two classes in the training cohort, and included them in the final feature set. After that, we performed feature selection using a random forest analysis and selected the top 15 proteins ranked by their mean decrease in accuracy (Fig. 5*B*). Subsequently, we randomly grouped the 15 protein features into 32,767 protein panels. To identify the best protein panels, we partitioned the samples from the training cohort into an inner training set (80%) and a validation set (20%) and evaluated the discriminatory ability of these panels to distinguish HCC from CCA. Notably, a three-protein panel (APOE, PKLR, and GALK1) exhibited exceptional performance, and we trained a final model based on these three proteins (APOE, PKLR, and GALK1). Finally, we used an independent validation cohort to evaluate the model's performance.

This three-protein classification model reached an AUC of 0.98 and an accuracy of 1 in the training cohort with sensitivity, specificity, positive predictive value (PPV), and negative predictive value (NPV) of 0.83, 1, 1, and 0.95, respectively (Fig. 5, *A*, *C*, *D*, and *G*).

### Independent Validation of the Three-Protein Classifier

To evaluate how well our model can be generalized, we collected an independent validation cohort, including 34 patients with HCC and 17 with CCA. Considering the differences between our training and validation cohorts regarding sample storage methods (FF tissues for the training cohort and FFPE tissues for the validation cohort) and MS instrumentation (QE-HF DIA for the training cohort and SCIEX5600 SWATH for the validation cohort), we assessed the concordance between the training and the validation cohorts at the protein and pathway levels. At protein level, 85% of proteins, that is, 5241, in the validation cohort could also be quantified in the training cohort (supplemental Fig. S3*A*). These overlapped proteins exhibited similar expression patterns between training and validation cohorts in cases with HCC and cases with CCA, respectively (supplemental Fig. S3*B*). Further differential protein analysis showed that the two cohorts shared 366 up-regulated proteins in common, contributing to about 81% of the up-regulated proteins of the validation cohort (supplemental Fig. S3, *C*–*F*). Regarding pathways identified, the results of our whole-proteome-based pathway enrichment using GSEA (supplemental Fig. S4, *A* and *B*) and our differentially expressed protein-based pathway enrichment using Metascape (supplemental Fig. S5) both showed that the pathways identified from each of the two cohorts were highly consistent.

Next, we assessed the model performance using the validation cohort. The AUC value of the model based on the validation set was 0.92 (Fig. 5*E*). A total of 46 (90%) out of the 51 samples in the validation cohort were correctly classified, with sensitivity, specificity, PPV, and NPV of 0.89, 0.94, 0.89, and 0.94, respectively (Fig. 5, *A*, *F*, and *G*).

The three proteins used in this model showed an almost identical expression pattern in both cohorts (Fig. 5*G*). Besides, the immunohistochemical (IHC) validation of these three proteins in patients with HCC or CCA, as obtained from the Human Protein Atlas database (supplemental Table S5), also showed similar trend, especially to proteins APOE and PKLR (38). Notably, APOE and PKLR are also part of the 73 key proteins distinguishing HCC, CCA, and benign tissues. Apolipoprotein E (APOE) is a major apoprotein of the chylomicron and is involved in the lipoprotein-mediated lipid transport between organs *via* the plasma and the interstitial fluids. It has been reported that the expression of APOE positively correlates with the HCC tumor grade (39). In our dataset, APOE was found to be up-regulated in HCC tumor tissues and down-regulated in the CCA tumor tissues (compared with benign tissues), suggesting important differences in lipid metabolism between HCC and CCA. Furthermore, APOE had the highest value of the mean decrease in accuracy (Fig. 5*A*), further corroborating its potential in distinguishing HCC from CCA. Pyruvate kinase L/R (PKLR) catalyzes the rate-limiting step of glycolysis, in which the phosphoenolpyruvate is converted into pyruvate and ATP. It has been reported that PKLR is a regulator of lipid metabolism and mitochondrial functions, and the inhibition of PKLR can reduce glucose uptake and mitochondrial activity in HepG2 cells (40). Our previous study also showed that pyruvate kinase M (PKM) is up-regulated in patients with HCC having chronic HBV infection (41). In this study, APOE and PKLR were up-regulated in HCC tumors compared with CCA tumors, indicating a substantial difference in lipid metabolism between HCC and CCA. The third protein used in our model was Galactokinase 1 (GALK1), which participates in the Leloir pathway, a critical step of galactose metabolism, by phosphorylating α-D-galactose into α-D-galactose 1-phosphate. In our data, the upregulation of GALK1 in the HCC tumor tissues suggested a galactose metabolic disorder, which was reported in rare congenital disorders, despite its role in tumor biology is not well understood (42). Manshu *et al.* (43) showed that siRNA targeting GALK1 or GALT can inhibit the growth of HepG2 cells, suggesting that GALK1 could be a therapeutic target against HCC.

### Abnormal Lipid Metabolism Characterized in the HCC Proteome Landscape

Lipids are essential components of cell membranes and other structural units of cells. In addition, lipids are used for energy storage and metabolism and play an important role as signaling molecules in various cellular processes. The regulation of lipid metabolism, such as lipid uptake, synthesis, and hydrolysis, is essential for maintaining the environmental homeostasis of the cell. The dysregulation of lipid metabolism is one of cancer's most prominent metabolic changes (44). Due to the need for rapid proliferation and the relative lack of external blood supply, HCC cells are often in a metabolic stress state due to hypoxia and insufficient nutrient supply. Consequently, HCC cells undergo a series of metabolic reprogramming steps to adapt to the harsh environment. In particular, lipid metabolic reprogramming is an important way for HCC cells to cope with metabolic stress (45).

In both our training and validation cohorts, the most significantly regulated pathways in the HCC tumor tissues were lipid metabolism-related (supplemental Fig. S4*B*). Proteins associated with lipid metabolism were up-regulated in HCC compared with CCA, including ACACB, SCD, PCCA, PCCB, AKR1C4, MMUT, SCP2, AKR1C3, FASN, and AMACR (supplemental Fig. S4*C*). The inhibition of fatty acid synthase (FASN) reduces fatty acid synthesis and leads to the subsequent cell cycle arrest and tumor cell apoptosis (46). Although FASN deprivation leads to the inhibition of HCC development in mice, it does not interfere with CCA development, further confirming that FASN is involved in the development of HCC but not CCA (47). In fact, the FASN inhibitor TVB-2640 is currently in Phase II clinical trials (48). Lastly, alpha-methylacyl-CoA racemase (AMACR) is located in mitochondria and peroxisomes and plays an essential role in bile acid biosynthesis and branched-chain fatty acid β-oxidation (49). Also, it has been shown that AMACR may be a prognostic indicator for predicting early recurrence/metastasis of HCC after hepatectomy (50). Several other proteins and their role in lipid metabolism are described in the Supplemental Information.

### The ECM-Related Pathway Characterized in the CCA Proteome Landscape

The ECM is a molecular substance secreted by cells into the extracellular stroma. It forms a complex network that supports and connects tissue structures and regulates tissue genesis and the physiological activities of cells. The occurrence, development, invasion, and metastasis of malignant tumors are often accompanied by changes in the expression of the ECM and its cell surface receptors (34). In our dataset, ECM-related pathways were significantly activated in the CCA tumor tissues. S100 calcium-binding protein A6 (S100A6), which was up-regulated in our CCA data, is involved in the transmission of cellular calcium signals and plays an important role in actin cytoskeleton reorganization and cell motility (supplemental Fig. S4*C*). It has been reported that S100A6 mRNA expression and protein levels are significantly increased in CCA, providing a valuable marker for distinguishing HCC from CCA (51). In addition, it has been shown that an increased S100A6 expression promotes cell proliferation and migration in HCC (52, 53). The enrichment of ECM-related pathways suggests that patients with CCA may be more prone to tumor metastasis and have a poorer prognosis than patients with HCC.

## Conclusion

In this work, we explored the proteome landscapes of two types of primary liver cancers: HCC and CCA. We found that 87% of the significantly regulated proteins in HCC were up-regulated in tumors compared to benign tissues. The regulated proteins' pathways were mainly associated with transcription, mRNA translation regulation, and post-translational modifications. On the other hand, 97% of significantly regulated proteins in CCA were down-regulated. The pathways involving the regulated proteins were mainly related to liver metabolic processes. The dysregulation of lipid metabolism is the most prominent feature of HCC tumors, and the enrichment of ECM-related pathways is the distinctive feature of CCA tumors. We finally trained a three-protein-based machine learning model to distinguish HCC from CCA, which achieved an AUC and accuracy of 0.92 and 0.90, respectively, using an independent validation cohort. This study has some limitations. Firstly, because of the relatively smaller sample size, there was no significant difference in protein expression between benign and malignant tissues in MCA cases. Secondly, the current DIA approach may not be sensitive enough to detect very low abundant proteins, thus it will likely identify more up-regulated proteins. Third, we analyzed the proteome of bulk tissues, ignoring the tumor heterogeneity. Emerging spatial proteomics and single-cell proteomics technologies are expected to shed light on tumor proteome heterogeneity with enhanced granularity.

## Data Availability

All data are available in the article or the Supplemental Information. The proteomics data have been deposited to the ProteomeXchange Consortium (http://proteomecentral.proteomexchange.org) via the iProX partner repository with the dataset identifier PXD043265. The project data analysis codes are deposited on GitHub (https://github.com/guomics-lab/HCC).

## Supplemental Data

This article contains supplemental data.

## Conflict of interest

T. G. and Y. Z. are shareholders of Westlake Omics, Inc. W. L. and L. H. are employees of Westlake Omics, Inc. The remaining authors declare no competing interests.

## Supplemental Data

Supplemental Table S5

Supplemental Figure S1 Venn diagram showing the number of peptides (*A*) and proteins (*B*) identified by three DIA software tools. **Supplemental Figure S2**. *A*, volcano plots showing the proteins with abundance difference between tumor and benign tissues in MCA patients. *B*, visualizations of meta-analysis results based on the dysregulated proteins between the benign and the tumor tissues from 41 HCC and 12 CCA patients. *C*, enrichment network visualization of the dysregulated proteins from Figure 3, *A* and *C*. **Supplemental Figure S3**. **Dysregulated proteins between the HCC and the CCA tumor tissues in the training set and the validation set.***A*, the quantified proteins in both the training and validation cohorts were ranked based on their relative expression using Z-score normalization. *B*, Venn diagram showing overlapping proteins between training cohort and validation cohort. *C* and *D*, volcano plots showing the proteins with significant abundance differences between the HCC and the CCA tumor tissues in the training cohort (*C*) and the validation cohort (*D*). *E* and *F*, Venn diagrams showing the overlapping up-regulated (*E*) and down-regulated proteins (*F*) from (*C*) and (*D*). **Supplemental Figure S4**. **Proteomic difference between the HCC and the CCA tumor tissues.***A*, GSEA of the protein abundances identified the ten most enriched pathways specific to HCC and CCA using the validation set. *B*, integrated analysis of the enriched pathway using the training cohort (cohort 1) and the validation cohort (cohort 2) between HCC and CCA. *C*, eleven selected proteins that were significantly regulated in HCC tumor tissues compared to CCA tumor tissues, using the training cohort and the validation cohort. **Supplemental Figure S5**. **Pathway level differences between the HCC and the CCA tumor tissues.***A* and *B*, heatmap showing the differential expression of proteins from the five most enriched pathways specific to HCC using the training cohort (*A*) and the validation cohort (*B*). *C*, Venn diagram showing the overlapping proteins between the top five pathways specific to HCC in the training set and the validation cohort. *D*, heatmap showing the differential expression of proteins from the five most enriched pathways specific to CCA using the validation cohort.
