# Supplementary material for: Proteome Landscapes of Human Hepatocellular Carcinoma and Intrahepatic Cholangiocarcinoma
Source: Mol Cell Proteomics. 2023 Jun 22;22(8):100604. doi: 10.1016/j.mcpro.2023.100604 (PMC10413158; doi:10.1016/j.mcpro.2023.100604)
Supplement: Supplemental Information [file mmc1.docx]

**Supplementary Information of**

**Proteome Landscapes of Human Hepatocellular Carcinoma and Intrahepatic Cholangiocarcinoma**

Xiao Yi^1, 2#^, Jiang Zhu^3, 4#^, Wei Liu^5^, Li Peng^6^, Cong Lu^3, 4^, Ping Sun^7^, Lingling Huang^5^, Xiu Nie^6^, Shi’ang Huang^3, 4^, Tiannan Guo^1, 2*^, Yi Zhu^1, 2*^

^1^Center for ProtTalks, Westlake Laboratory of Life Sciences and Biomedicine, Key Laboratory of Structural Biology of Zhejiang Province, School of Life Sciences, Westlake University, Hangzhou, Zhejiang Province, China;

^2^Institute of Basic Medical Sciences, Westlake Institute for Advanced Study, Hangzhou, Zhejiang Province, China;

^3^ Center for Stem Cell Research and Application, Union Hospital, Tongji Medical College, Huazhong University of Science and Technology, Wuhan, Hubei, China;

^4^Key laboratory of Biological Targeted Therapy, the Ministry of Education, Union Hospital, Tongji Medical College, Huazhong University of Science and Technology, Wuhan, Hubei, China;

^5^ Westlake Omics (Hangzhou) Biotechnology Co, Ltd, Hangzhou, China;

^6^ Department of Pathology, Union Hospital, Tongji Medical College, Huazhong University of Science and Technology, Wuhan 430022, China

^7^ Department of Hepatobiliary Surgery, Union Hospital, Tongji Medical College, Huazhong University of Science and Technology, Wuhan, Hubei, China.

^#^ These authors contribute equally

^*^Correspondence: [guotiannan@westlake.edu.cn](mailto:guotiannan@westlake.edu.cn) (T.G.); [zhuyi@westlake.edu.cn](mailto:zhuyi@westlake.edu.cn) (Y.Z.)

In the main manuscript, we used 73 proteins to distinguish HCC tumor tissues from CCA tumor tissues and benign tissues. To better understand why these proteins were selected, we reviewed the top ten features (Figure 4C) based on their biological functions, as shown below.

Apolipoprotein E (APOE) is a major apoprotein of the chylomicron and is involved in the lipoprotein-mediated lipid transport between organs via the plasma and interstitial fluids. It has been reported that there is a positive correlation between the expression of APOE and the tumor grade of HCC [1]. In our dataset, APOE was up-regulated in the HCC tumor tissues and down-regulated in the CCA tumor tissues compared with benign tissues, suggesting differences in lipid metabolism between HCC and CCA. Asialoglycoprotein receptor 2 (ASGR2) is a subunit of the asialoglycoprotein receptor, which mediates the endocytosis of plasma glycoproteins and the lysosomal degradation of glycoproteins. An interference with asialoglycoprotein receptors enhances the inhibition of hepatitis B virus production [2]. In our dataset, ASGR2 was up-regulated in the HCC and benign tissues compared with CCA tissues. This may be related to the different pathogenesis of HCC and CCA, suggesting the need for different targeted therapies for the two groups.

The remaining eight proteins belong to cluster 2 (Figure 4A). They were down-regulated in the HCC and CCA tumors and up-regulated in the benign tissues. EH domain containing 3 (EHD3) is an ATP and membrane binding protein that controls membrane recombination/tubular shape during ATP hydrolysis and is related to the cholesterol and the sphingolipids transport pathways [3]. EHD3 was also reported to function as a tumor suppressor by controlling cell cycle arrest and apoptosis. In liver cancer cells, EHD3 likely regulates the proliferation of cancer cells through the same mechanism. However, further experiments are needed to confirm this hypothesis. Solute carrier family 22 member 1 (SLC22A1) belongs to a family of organic cation transport proteins essential for helping cells (especially in organs that perform metabolic functions, such as the liver and the kidneys) eliminate endogenous and environmental toxins. Aldehyde dehydrogenase 8 family member A1 (ALDH8A1) has a similar function to SLC22A1: it is responsible for the enzymatic detoxification of endogenous and exogenous aldehydes. Our previous study showed that multiple proteins of the ALDH family are down-regulated in HCC [4]. Cytochrome P450 family 4 subfamily A member 11 (CYP4A11) is a member of the cytochrome P450 superfamily of enzymes that perform cellular detoxification functions by metabolizing drugs, fatty acids, and their oxygen content derivatives. Studies have shown that CYP4A11 decreases in HCC, and its expression is a favorable prognostic factor for HCC [5, 6]. Aminocarboxymuconate semialdehyde decarboxylase (ACMSD) can divert alpha-amino-beta-carboxy-muconate-epsilon-semialdehyde (ACMS) to a benign catabolite and is associated with tryptophan catabolism, a key regulator of mammalian NAD+ metabolism [7]. Studies have pointed out that the ACMSD down-regulation is a potential mechanism of tumor progression and an important therapeutic target for brain cancer treatment [8, 9]. FGGY carbohydrate kinase domain-containing protein (FGGY) phosphorylates carbohydrates, preventing the toxic accumulation of free D-ribulose. The down-regulation of these proteins is responsible for detoxifying tumor tissues, protecting cells from toxicants that lead to cellular damage and further developing cellular carcinogenesis. The six-transmembrane epithelial antigen of the prostate 4 (STEAP4) and other family proteins (STEAP2 and STEAP3) are membrane metalloreductases that move electrons, from cytosolic NADPH to extracellular iron or copper. STEAP4 is involved in the cellular response to inflammatory stress and protects cells from damage [10]. C-Type lectin domain family 4 member M (CLEC4M) is a pathogen-recognition receptor capable of recognizing and mediating the endocytosis of many pathogens, including hepatitis C and HIV-1. It has been reported that the expression of CLEC4M is associated with the progression of HCC and the infiltration of various immune cells, providing evidence of a potential new immunotherapy target against HCC [11].

Comparing the training and validation cohorts, we found that the most significantly regulated pathways in HCC tumor tissue from both cohorts were the lipid metabolism-related pathways (Figure S4B). We then selected ten up-regulated features (Figure S4C) related to their biological functions in HCC when compared with CCA, as shown below. FASN and AMACR have been described in the manuscript and will not be repeated here.

Acetyl-CoA carboxylase beta (ACACB) and stearoyl-CoA desaturase (SCD) are essential enzymes involved in the biosynthesis of fatty acids [12]. Propionyl-CoA carboxylase subunit alpha (PCCA) and beta (PCCB) are mitochondrial enzymes that are related to the catabolism of odd chain fatty acids [13]. Aldo-keto reductase family 1 member C3 (AKR1C3) and C4 (AKR1C4) are cytosolic Aldo-keto reductases; they catalyze NADPH-dependent reductions and regulate the metabolism of androgens, estrogens, and progesterone; also, they are promising anti-cancer targets [14]. Methylmalonyl-CoA mutase (MMUT) catalyzes the isomerization of methylmalonyl-CoA to succinyl-CoA and plays an essential role in the tricarboxylic acid cycle. Sterol carrier protein 2 (SCP2) is a crucial factor in the peroxisomal oxidation of branched-chain fatty acids [15].

**Reference**

1. Peng, H., et al., *Serum and tissue proteomic signatures of patients with hepatocellular carcinoma using 2D gel electrophoresis.* Mol Med Rep, 2019. **20**(2): p. 1025-1038.

2. Eto, T. and H. Takahashi, *Enhanced inhibition of hepatitis B virus production by asialoglycoprotein receptor-directed interferon.* Nat Med, 1999. **5**(5): p. 577-81.

3. Lu, Q., et al., *Early steps in primary cilium assembly require EHD1/EHD3-dependent ciliary vesicle formation.* Nat Cell Biol, 2015. **17**(3): p. 228-240.

4. Zhu, Y., et al., *Identification of Protein Abundance Changes in Hepatocellular Carcinoma Tissues Using PCT-SWATH.* Proteomics Clin Appl, 2019. **13**(1): p. e1700179.

5. Chen, H., et al., *Expression of P450 and nuclear receptors in normal and end-stage Chinese livers.* World J Gastroenterol, 2014. **20**(26): p. 8681-90.

6. Eun, H.S., et al., *Cytochrome P450 4A11 expression in tumor cells: A favorable prognostic factor for hepatocellular carcinoma patients.* J Gastroenterol Hepatol, 2019. **34**(1): p. 224-233.

7. Palzer, L., et al., *Alpha-Amino-Beta-Carboxy-Muconate-Semialdehyde Decarboxylase Controls Dietary Niacin Requirements for NAD(+) Synthesis.* Cell Rep, 2018. **25**(5): p. 1359-1370 e4.

8. Guillemin, G.J., et al., *Characterization of the kynurenine pathway in human neurons.* J Neurosci, 2007. **27**(47): p. 12884-92.

9. Adams, S., et al., *The kynurenine pathway in brain tumor pathogenesis.* Cancer Res, 2012. **72**(22): p. 5649-57.

10. Scarl, R.T., et al., *STEAP4: its emerging role in metabolism and homeostasis of cellular iron and copper.* J Endocrinol, 2017. **234**(3): p. R123-R134.

11. Zhang, Y., et al., *CLEC4s as Potential Therapeutic Targets in Hepatocellular Carcinoma Microenvironment.* Front Cell Dev Biol, 2021. **9**: p. 681372.

12. Wang, J., et al., *Characterization of HSCD5, a novel human stearoyl-CoA desaturase unique to primates.* Biochem Biophys Res Commun, 2005. **332**(3): p. 735-42.

13. Stankovics, J. and F.D. Ledley, *Cloning of functional alpha propionyl CoA carboxylase and correction of enzyme deficiency in pccA fibroblasts.* Am J Hum Genet, 1993. **52**(1): p. 144-51.

14. Zeng, C.M., et al., *Aldo-Keto Reductase AKR1C1-AKR1C4: Functions, Regulation, and Intervention for Anti-cancer Therapy.* Front Pharmacol, 2017. **8**: p. 119.

15. Ferdinandusse, S., et al., *Peroxisomal fatty acid oxidation disorders and 58 kDa sterol carrier protein X (SCPx). Activity measurements in liver and fibroblasts using a newly developed method.* J Lipid Res, 2000. **41**(3): p. 336-42.
