# Supplementary material for: Proteome Landscapes of Human Hepatocellular Carcinoma and Intrahepatic Cholangiocarcinoma
Source: Mol Cell Proteomics. 2023 Jun 22;22(8):100604. doi: 10.1016/j.mcpro.2023.100604 (PMC10413158; doi:10.1016/j.mcpro.2023.100604)
Supplement: Supplemental Table S1 [file mmc2.docx]

| **Characteristics** | **Training cohort** | | | **Validation cohort** | |
| --- | --- | --- | --- | --- | --- |
|  | **HCC (N=41)** | **CCA (N=12)** |  | **HCC (N=34)** | **CCA (N=17)** |
| **Gender (%)** |  |  |  |  |  |
| **Male** | **35 (85.4)** | **8 (66.7)** |  | **30 (88.2)** | **13 (76.5)** |
| **Female** | **6 (14.6)** | **4 (33.3)** |  | **3 (8.8)** | **1 (5.9)** |
| **Unknown** | **0 (0.0)** | **0 (0.0)** |  | **1 (2.9)** | **3 (17.6)** |
| **Age, median** | **53 (38, 70)** | **55 (33, 70)** |  | **53** | **53 (40, 71)** |
| **Age, years n (%)** |  |  |  |  |  |
| **＜50** | **18 (43.9)** | **2 (16.7)** |  | **11 (32.4)** | **5 (29.4)** |
| **50-64** | **14 (34.1)** | **8 (66.7)** |  | **16 (47.1)** | **8 (47.1)** |
| **≥65** | **9 (22.0)** | **2 (16.7)** |  | **6 (17.6)** | **1 (5.9)** |
| **Unknown** | **0 (0.0)** | **0 (0.0)** |  | **1 (2.9)** | **3 (17.6)** |

**Table S1. Demographics of the patients in training cohort and validation cohort**
