# Supplementary figures and images for: Proteome Landscapes of Human Hepatocellular Carcinoma and Intrahepatic Cholangiocarcinoma
Source: Mol Cell Proteomics. 2023 Jun 22;22(8):100604. doi: 10.1016/j.mcpro.2023.100604 (PMC10413158; doi:10.1016/j.mcpro.2023.100604)

Figure S1

A

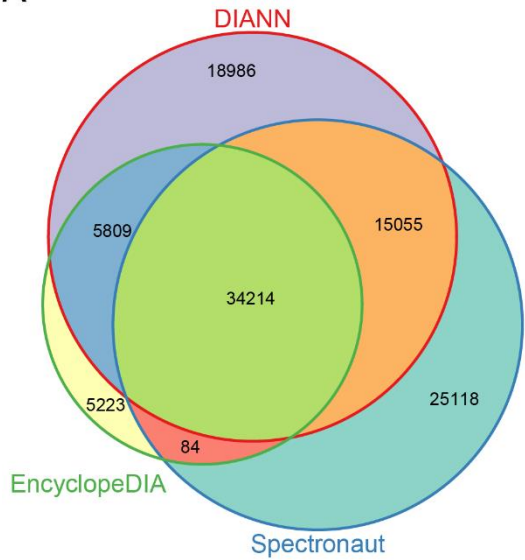

B

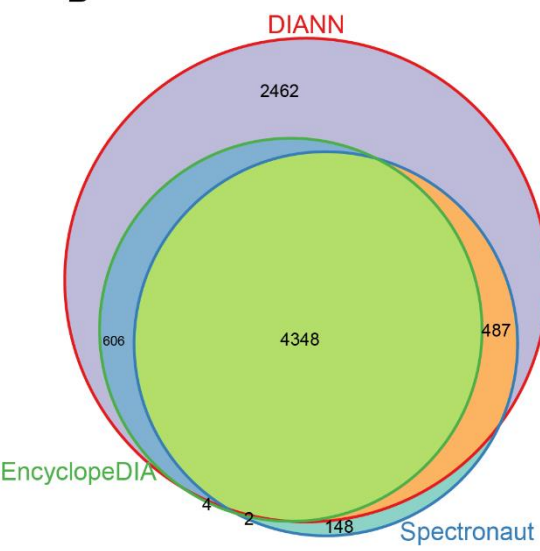

Figure S2

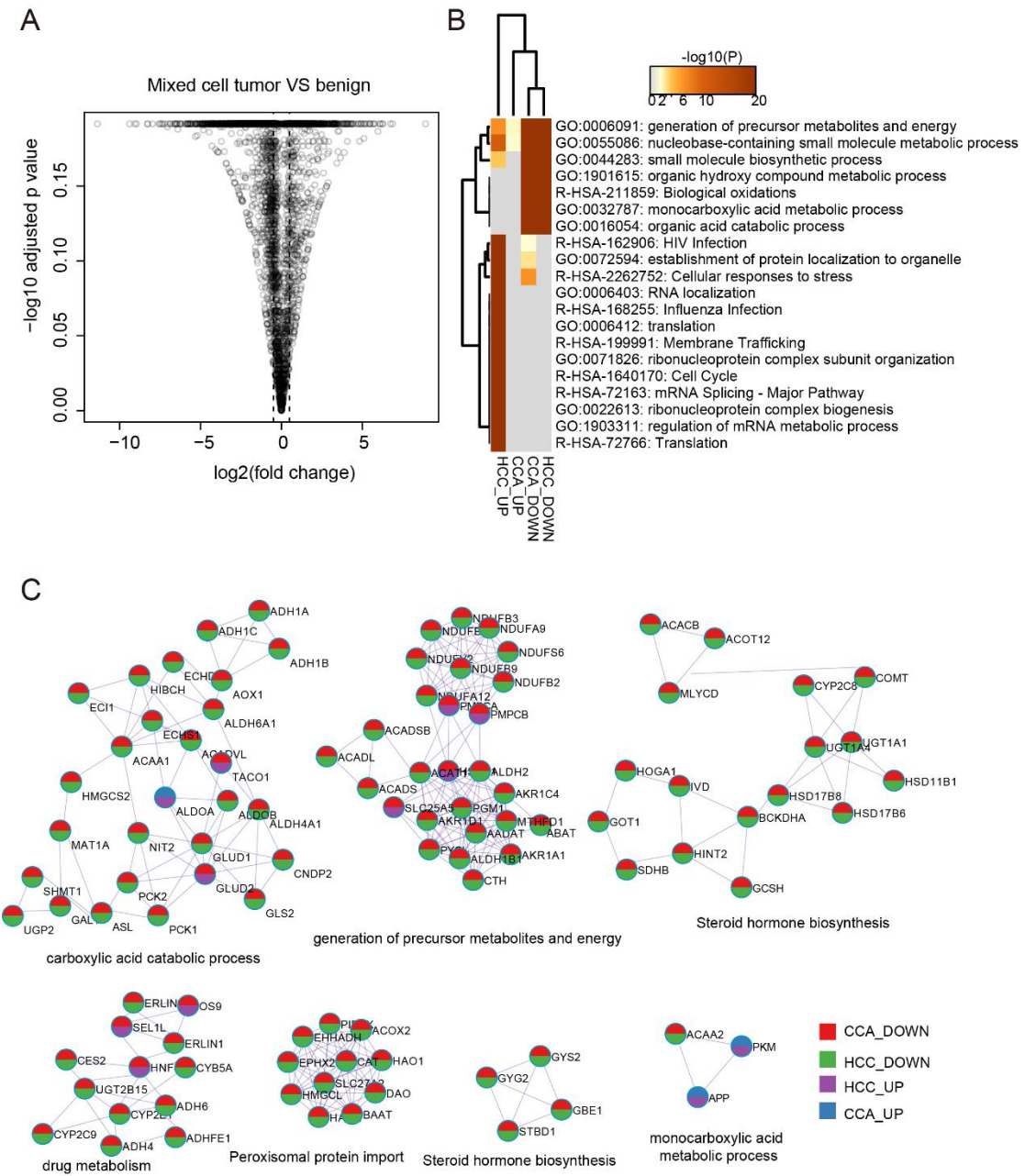

Figure S3

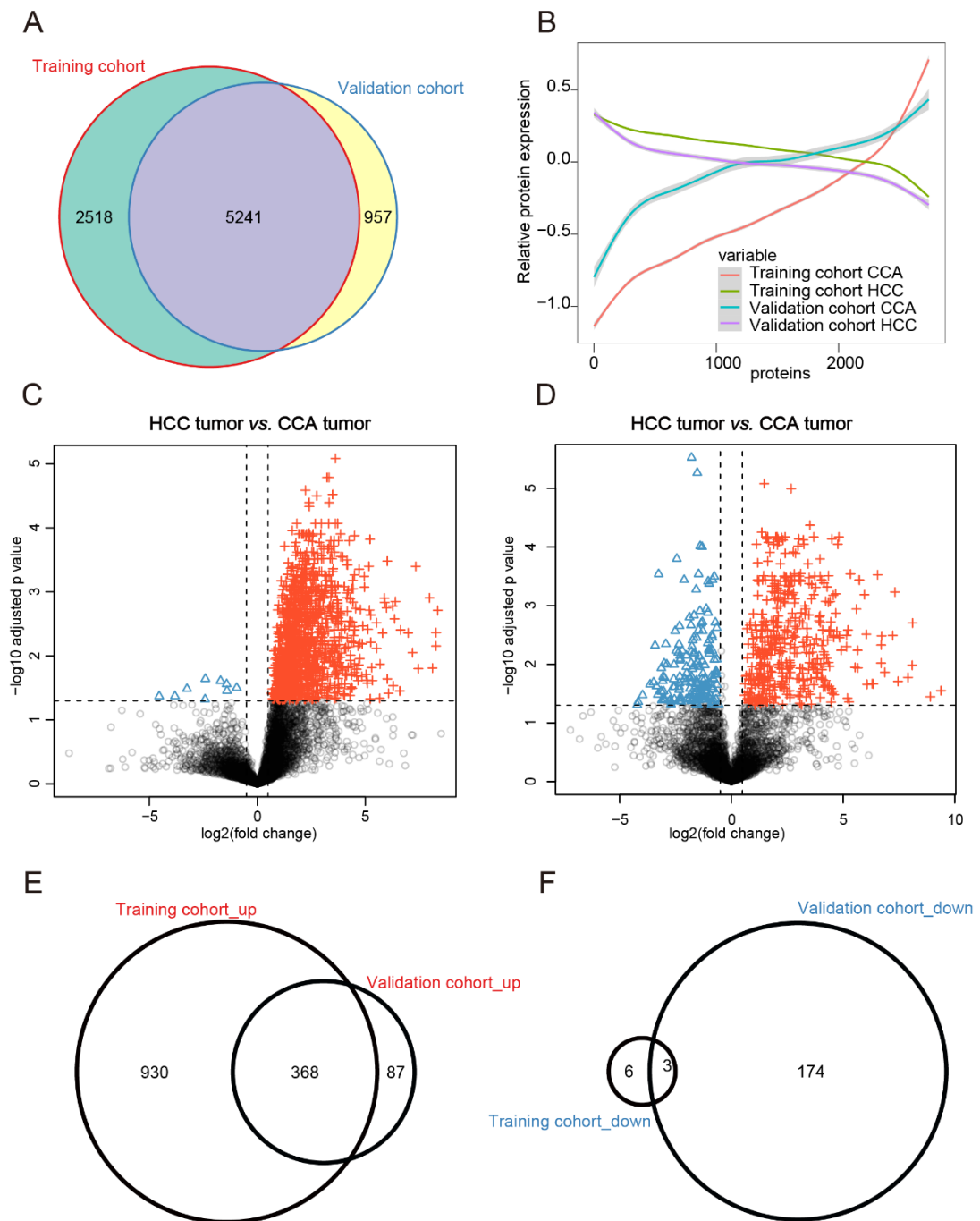

Figure S4

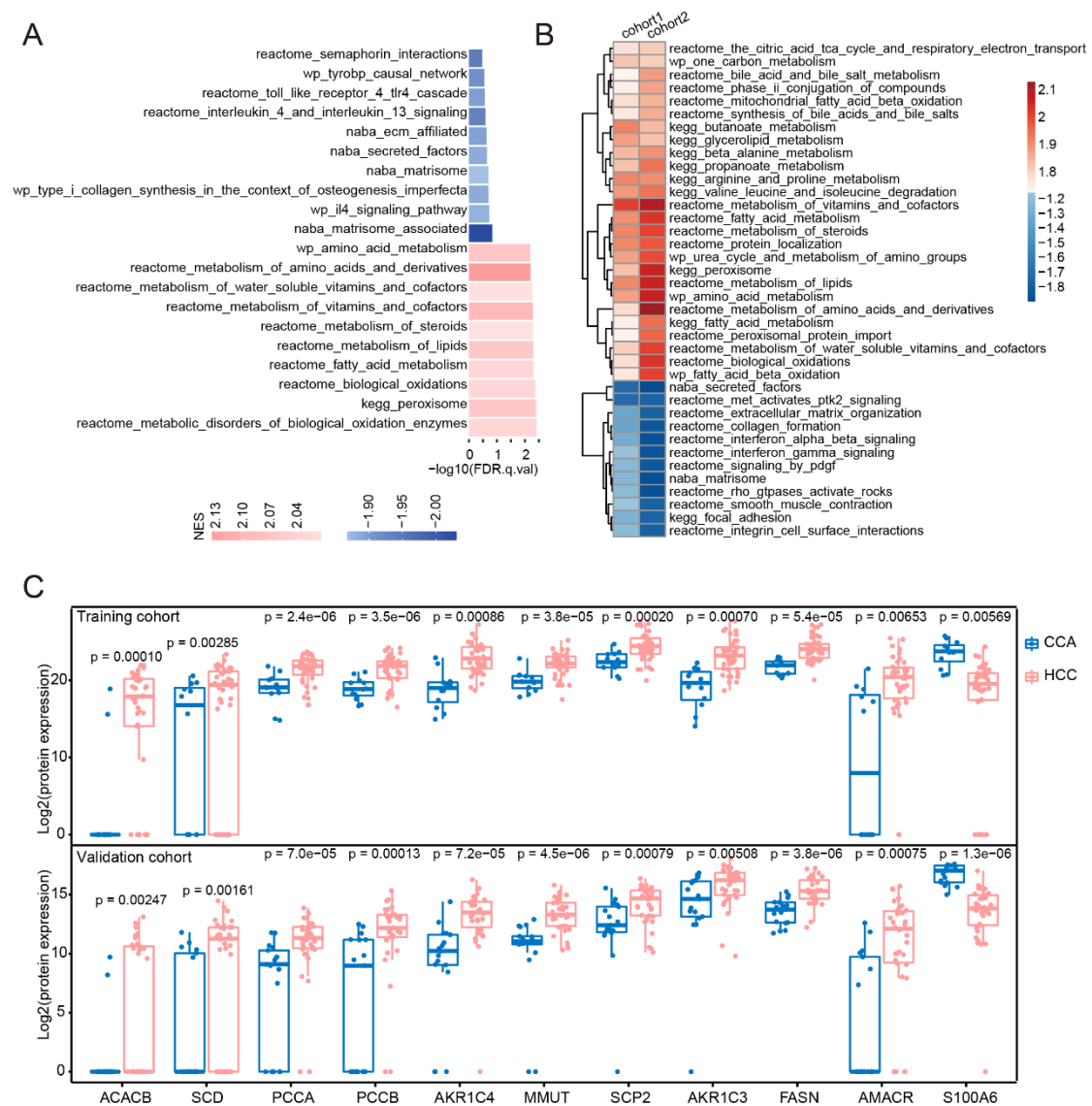

Figure S5

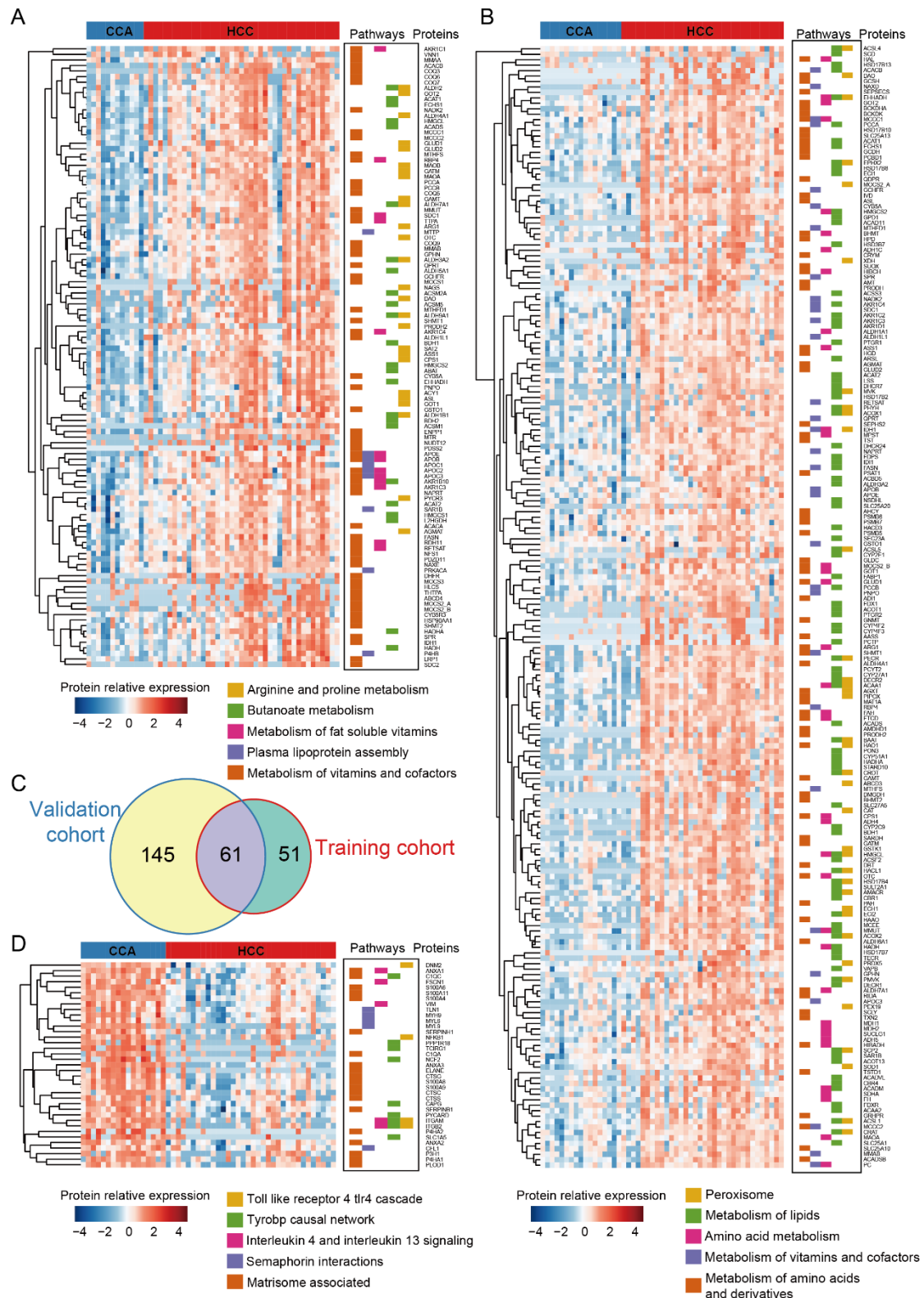

Supplement: Supplemental Figure S1 — Venn diagram showing the number of peptides (A) and proteins (B) identified by three DIA software tools. Supplemental Figure S2. A, volcano plots showing the proteins with abundance difference between tumor and benign tissues in MCA patients. B, visualizations of meta-analysis results based on the dysregulated proteins between the benign and the tumor tissues from 41 HCC and 12 CCA patients. C, enrichment network visualization of the dysregulated proteins from Figure 3, A and C. Supplemental Figure S3. Dysregulated proteins between the HCC and the CCA tumor tissues in the training set and the validation set.A, the quantified proteins in both the training and validation cohorts were ranked based on their relative expression using Z-score normalization. B, Venn diagram showing overlapping proteins between training cohort and validation cohort. C and D, volcano plots showing the proteins with significant abundance differences between the HCC and the CCA tumor tissues in the training cohort (C) and the validation cohort (D). E and F, Venn diagrams showing the overlapping up-regulated (E) and down-regulated proteins (F) from (C) and (D). Supplemental Figure S4. Proteomic difference between the HCC and the CCA tumor tissues.A, GSEA of the protein abundances identified the ten most enriched pathways specific to HCC and CCA using the validation set. B, integrated analysis of the enriched pathway using the training cohort (cohort 1) and the validation cohort (cohort 2) between HCC and CCA. C, eleven selected proteins that were significantly regulated in HCC tumor tissues compared to CCA tumor tissues, using the training cohort and the validation cohort. Supplemental Figure S5. Pathway level differences between the HCC and the CCA tumor tissues.A and B, heatmap showing the differential expression of proteins from the five most enriched pathways specific to HCC using the training cohort (A) and the validation cohort (B). C, Venn diagram showing the overlapping proteins between [file mmc7.pdf]
